# Skin calcium deposits in primary familial brain calcification: A novel potential biomarker

**DOI:** 10.1002/acn3.52304

**Published:** 2025-02-11

**Authors:** Aron Emmi, Giulia Bonato, Aleksandar Tushevski, Cinzia Bertolin, Francesco Cavallieri, Andrea Porzionato, Angelo Antonini, Leonardo Salviati, Miryam Carecchio

**Affiliations:** ^1^ Institute of Human Anatomy, Department of Neurosciences University of Padova Padova 35129 Italy; ^2^ Parkinson and Movement Disorders Unit, Department of Neuroscience, Centre for Rare Neurological Diseases (ERN‐RND) University of Padova Padova 35128 Italy; ^3^ Center for Neurodegenerative Disease Research (CESNE) University of Padova Padova 35128 Italy; ^4^ Clinical Genetics Unit University Hospital of Padova Padova 35128 Italy; ^5^ Neurology Unit, Neuromotor and Rehabilitation Department Azienda USL‐IRCCS di Reggio Emilia Reggio Emilia 42123 Italy; ^6^ Clinical Genetics Unit, Department of Women's and Children's Health University of Padova Padova 35128 Italy

## Abstract

**Objective:**

Primary Familial Brain Calcification (PFBC) is a rare neurodegenerative disorder characterized by small vessel calcifications in the basal ganglia. PFBC is caused by pathogenic variants in different genes and its physiopathology is still largely unknown. Skin vascular calcifications have been detected in single PFBC cases, suggesting that calcium deposition may not be limited to the brain, but it is unknown whether this is a hallmark of all PFBC genetic and clinical subtypes. This work aims at assessing anatomical and subcellular localization of calcium‐phosphate deposits in skin biopsies from PFBC patients to ascertain the accuracy of histological calcium staining in differentiating PFBC from healthy controls (HC) and Parkinson's Disease (PD).

**Methods:**

Histopathology and light microscopy of skin biopsy from 20 PFBC, 7 HC and 10 PD subjects (3 mm ø–5 mm deep punch biopsies, Hematoxylin–Eosin and vonKossa staining, immunoperoxidase CD31 staining); clinical, genetic and radiological assessment.

**Results:**

Unlike HC and PD subjects, the majority of PFBC patients (17/20) showed a consistent pattern of granular argyrophilic calcium‐phosphate deposits in the basal lamina and the cytoplasm of CD31+ endothelial cells and pericytes of dermal capillaries, and the basement membrane of sweat glands. This pattern was unrelated to the underlying mutated gene or clinical status.

**Interpretation:**

Skin biopsy may be a novel PFBC diagnostic tool and a potential biomarker for future therapies, and a tool to investigate PFBC disease mechanisms. Different findings in some patients could be due to skin sampling variability and biological consequences of specific PFBC gene variants.

## Introduction

Primary Familial Brain Calcification (PFBC), formerly known as Fahr's disease or idiopathic basal ganglia calcification, is a rare neurodegenerative disorder characterized by calcification of small brain vessels, affecting the basal ganglia and other brain regions including thalami, cerebellar dentate nuclei, subcortical white matter, brainstem and cerebral cortex.^1^

PFBC is a genetic disease caused by autosomal dominant (AD) and recessive (AR) genes discovered since 2012. Dominantly inherited PFBC is associated with pathogenic variants in *SLC20A2* (solute carrier 20 member 2),^2^ *XPR1* (xenotropic and polytropic retrovirus receptor 1),^3^ *PDGFB* (platelet‐derived growth factor B)^4^ and *PDGFRB* (platelet‐derived growth factor receptor B).^5^ Recessively inherited PFBC is caused by *MYORG* (Myogenesis Regulating Glycosidase protein),^6^ *JAM2* (Junctional Adhesion Molecule 2)^7^ and *NAA60* (N‐Alpha‐Acetyltransferase 60).^8^ The recently discovered *CMPK2* gene (cytidine monophosphate – UMP‐CMP kinase 2)^9^ has been associated with basal ganglia calcification in two subjects, but its causative role in PFBC is still debated.

Clinical manifestations of PFBC are highly heterogeneous and include hypo‐ and hyperkinetic movement disorders (most frequently parkinsonism), cognitive impairment and psychiatric symptoms, with significant intra‐ and inter‐familial phenotypic heterogeneity. Patients with radiological evidence of cerebral calcification may not show signs or symptoms of the disease even in late adulthood.^10^ In fact, pathogenic variants in PFBC‐related genes are fully penetrant radiologically, but they show reduced clinical penetrance, with some differences related to the specific underlying mutated gene.^11^, ^12^ The characteristic neuropathological alteration in PFBC is the presence of calcium‐phosphate deposits and nodules along the capillaries of the brain vasculature of specific anatomic regions, especially the basal ganglia. The pathophysiological mechanisms leading to calcium deposition and neurodegeneration in PFBC are still largely unknown, but a central role of the neurovascular unit (NVU) has emerged in recent years. The NVU is the microanatomic structure constituting the blood–brain barrier (BBB), that controls the trafficking of blood‐derived molecules and cells into the central nervous system (CNS).^13^

Pericytes are capillary wall cells embedded in the endothelial basement membrane and have an important role in brain vasculature development during embryogenesis and in the regulation of BBB during adult life.^14^, ^15^ An altered function of pericytes at the NVU has been demonstrated in animal models of PFBC and is now thought to have a central role in its pathogenesis.^16^

It is thought that pathogenic variants in PFBC‐related genes, through different pathways, lead to a dysfunction of the NVU, pericyte deficiency and altered properties of the endothelial cells, leading to a progressive ossification of the NVU and neurotoxicity with chronic neurodegeneration, as reflected by the progressive nature of this condition.^17^

PFBC has been traditionally regarded as a primary brain disorder, as opposed to systemic diseases characterized by abnormal calcifications also in other organs or endocrinological conditions such as hypoparathyroidism that can lead to cerebral calcifications as a result of an altered peripheral phosphor‐calcium metabolism. For this reason, the diagnostic workup to formulate a diagnosis of PFBC generally does not include non‐neurological diagnostic procedures.

Skin biopsies performed in three PFBC patients carrying variants in *PDGFB*, *PDGFRB* and *XPR1* genes, respectively, showed the presence of microangiopathy and microcalcifications in the basal lamina.^18^, ^19^ This finding suggests that, while manifesting predominantly as a neurological disorder, extra‐cerebral involvement in PFBC may represent an under‐valued aspect of the disease, similar to alpha‐synuclein deposition in the skin of Parkinson's disease (PD) patients.^20^ Furthermore, skin biopsy may represent an additional minimally invasive tool for diagnostic purposes and a potential marker to assess the efficacy of future therapies to chelate calcium or modify disease progression.

Currently, it is unknown whether microangiopathy and peripheral vessel calcifications are a common feature of PFBC or are associated with variants in specific genes, disease duration or disease severity. Skin biopsies from three PDGFB variant carriers from a Swedish family did not show major alterations in subcutaneous capillary vessels, but methods used (hematoxylin and eosin staining and electron microscopy) were not specific to detect capillary calcification and might have overlooked pathological changes.^21^

The aim of this study was to evaluate the anatomical and subcellular localization of calcium‐phosphate deposits in skin biopsies of PFBC patients with different mutated genes and clinical features via histopathology and light microscopy and to ascertain the accuracy of histological calcium staining in differentiating PFBC from healthy controls and other neurodegenerative conditions affecting the basal ganglia, such as Parkinson's Disease.

## Materials and Methods

Twenty patients diagnosed with PFBC (9 males, 11 females) from 17 different families, followed by the Department of Neurology of Padua University, were included in the study. All patients consented to undergo skin biopsies and genetic testing. The study was conducted in accordance with the Declaration of Helsinki and Ethics Guidelines from the home Institution, as part of a biocollection programme for different neurodegenerative disorders and approved by our Ethical Commitee; all patients signed a dedicated written informed consent form for all study procedures and data sharing.

The control group included two subgroups: (1) Ten patients diagnosed with idiopathic PD (7 males, 3 females, mean age 66.3 years; mean disease duration 11.4 years), without evidence of variants in known PD‐associated genes (PD group); and (2) seven healthy subjects (4 males, 3 females; mean age 60.5 years) with no family history of neurological or psychiatric diseases and normal CT scan (performed for reasons unrelated to the present study, e.g., minor head trauma; HC group).

CT scan was available in all PFBC patients, who underwent a full neurological examination and a neuropsychological battery to assess cognitive functions. Secondary causes of brain calcifications including parathyroid dysfunction were ruled out before genetic studies.

Genetic testing was performed with Next Generation Sequencing Illumina Nextseq550 platform kit Agilent Suserelect analyzing a custom‐made panel including more than 100 genes related to movement disorders (including Parkinson's disease) and PFBC‐related genes (see Supplementary Material for gene list). For each gene, the entire coding region and intron‐exon junctions ±10 nucleotides were covered with a sequencing depth of at least 20x for over 99% of the target region. The reference sequence for alignment and comparison was GRCh37 (Genome Reference Consortium Human Build 37). Variants were classified according to the ACMG‐AMP (American College of Medical Genetics and Genomics—Association for Molecular Pathology) guidelines^22^ and phenotype; publicly available variants databases (GnomAD) and *in silico* prediction tools (Sift, PolyPhen, MutationTaster) were consulted for variant interpretation. Segregation analysis (including evidence of calcification on brain CT scan to determine the relatives' affected/non‐affected status) was performed when possible.

3 mm ø – 5 mm deep punch biopsies of the skin at the level of the deltoid muscle were performed for each subject included. Sampled biopsies were fixed overnight at 4°C in Zamboni solution, processed for paraffin‐embedding and sectioned at the rotary microtome (Leica RM2155) into 5 μm thick sections. Hematoxylin and Eosin staining was employed for routine histopathological evaluation. The von Kossa method for histological staining of calcium phosphate was employed to evaluate skin calcium deposits.

Immunoperoxidase staining was performed on a Dako EnVision Autostainer (Dako Denmark A/S, Glostrup, Denmark) according to manufacturer recommendations and as previously reported.^23^ CD31/PECAM‐1 (monoclonal mouse anti‐Human, Clone 390, 1:50, eBioScience #14–0311‐82) was used to evaluate endothelial cells and pericytes; von Kossa staining was performed on the same sections prior to CD31 immunoperoxidase in order to evaluate argyrophilic grain colocalization. Antigen retrieval was performed on a PT‐Link Dako Antigen retrieval station using a Citrate buffer at pH 6 solution at 96° for 15 min. Slides were evaluated by an experienced pathologist on a Leica LMD6 light microscopy station.

## Results

PFBC patients' mean age was 56.3 years, ranging from 39 to 70 years, with a mean disease duration in clinically affected subjects of 6.2 years (ranging from 1 to 16 years). Family history was positive for PFBC in 9 patients and for dementia in one subject, but without available brain imaging of the affected family member. Patients no. 2 and 12 belonged to the same family, as well as no. 3 and 4; all other patients were unrelated. 13 patients were clinically symptomatic and 7 were asymptomatic, including a formal neuropsychological assessment that did not reveal cognitive decline. Parkinsonism was observed in 7 symptomatic subjects, being the most frequent finding on examination. One patient had Levodopa‐responsive asymmetric parkinsonism mimicking PD, whereas in five patients bradykinesia was associated with cerebellar signs (dysarthria and gait ataxia) and one patient presented a combination of dystonia and parkinsonism with pyramidal signs and cognitive decline. Paroxysmal dystonia was the only clinical manifestation in 2 subjects. Neuropsychiatric features were documented in 13 subjects, of whom eight had mild cognitive impairment (MCI). Pyramidal signs (brisk reflexes) were observed in 12 patients, including three asymptomatic subjects.

CT scans showed extensive bilateral basal ganglia involvement in all subjects; calcium deposition was observed in the thalami in 15/20 cases, in cerebellar dentate nuclei in 16/20, in subcortical white matter in 10/20; cortical calcifications in the parietal and occipital lobes were found in 2 subjects carrying biallelic *MYORG* variants, one of whom also had central pontine calcifications. The mean total calcification score (TCS^24^) in the PFBC group was 26.4.

All but two patients had variants in known PFBC‐related genes; 7 carried variants in *SLC20A2* gene, 8 in *MYORG* (1 with a homozygous variant – Pat. no. 5 –, 4 with two compound heterozygous variants – Pat. no. 7, 17, 18 and 19 –, 2 siblings with three variants, 2 with an *in cis* and one with an *in trans* configuration – Pat. no. 2 and 12 –, and one patient with 2 *in‐cis* homozygous variants – Pat. no. 14 –), one in *PDGFB*, one in *PDGFRB* and one in *XPR1*.

With regard to the phase of *MYORG* variants, this was determined by segregation analysis when relatives were available (Pat no. 2, 5, 7, 12 and 17). In Patient 18 parents were not available but his brother, who displayed extensive cerebral calcification and suffered from severe psychiatric disturbances, carried the same *MYORG* variants, thus suggesting a pathogenic role of them, even though full segregation status analysis remains incomplete. The only *MYORG* patient with no relatives available was Patient 19 but clinical and radiological features were consistent with a diagnosis of PFBC. Patient 14 carries two missense *MYORG* variants apparently in a homozygous state. Since they are the same two variants found with an in‐cis configuration in patients 2 and 12, this suggests that this could be a rare haplotype with a complex allele, that Patient 14 (not related to Pt. 2 and 12) has in homozygous state and patients 2 and 12 have in compound heterozygosity with a frameshift variant.

Out of 23 variants found, 19 were missense and 4 frameshift variants leading to a premature stop codon. Five variants were previously reported in the literature,^25^, ^26^, ^27^, ^28^, ^29^, ^30^ whereas all the other variants were novel and classified as likely pathogenic (ACMG class 4, Table S1) based on allele frequency, *in silico* prediction tools and clinical‐radiological features of carriers; segregation analysis further supported a pathogenic role of these variants where available.

Patients' clinical and genetic data are reported in Tables 1 and 2.

**Table 1:** PFBC patients' genetic data.

Patient No.	Gene	Variant 1	Variant 2	Het/Hom	Segregation analysis performed	Proband/family member	Novel variant or reference
1	*PDGFB*	NM_002608.2:c.298C>T p.(Arg100Cys)	–	Het	Yes	P	Shen *et al*. 2022 [25]
2	*MYORG*	NM_020702.3:c.686G>A p.(Trp229*)	NM_020702.3:c.[1318G>A; 1189T>A] p.[(Ala440Thr); (Tyr397Asn)]	Comp‐Het	Yes	P	Novel
3	*SLC20A2*	NM_006749.4:c.84T>A p.(Asp28Glu)	–	Het	Yes	P	Novel
4	*SLC20A2*	NM_006749.4:c.84T>A p.(Asp28Glu)	–	Het	Yes	F (Pat. no. 3)	Novel
5	*MYORG*	NM_020702.3:c.1270_1277dup p.(Trp426Cysfs*11)	–	Hom	Yes	P	Chelban *et al*. 2020 [26]
6	*SLC20A2*	NM_001257180.2:c.212G>A p.(Arg71His)	–	Het	No	P	Yamada *et al*. 2014; Ramos *et al*. 2020 [27, 28]
7	*MYORG*	NM_020702.3:c.1321del p.(Arg441Alafs*65)	NM_020702.3:c.1841T>C p.(Leu614Pro)	Comp‐Het	Yes	P	Novel
8	Unknown	–	–	–	–	P	–
9	*XPR1*	NM_004736.4:c.194A>G p.(Glu65Gly)	–	Het	No	P	Novel
10	*PDGFRB*	NM_002609.3:c.2051G>T p.(Cys684Phe)	–	Het	No	P	Novel
11	*SLC20A2*	NM_001257180.2:c.1472A>G p.(Gln491Arg)	–	Het	Yes	P	Novel
12	*MYORG*	NM_020702.3:c.686G>A p.(Trp229*)	NM_020702.3:c.[1318G>A;1189T>A] p.[(Ala440Thr);(Tyr397Asn)]	Comp‐Het	Yes	F (Pat. no. 2)	Novel
13	*SLC20A2*	NM_006749.4:c.541C>T p.(Arg181Trp)	–	Het	Yes	P	Lamquet *et al*. 2019 [29]
14	*MYORG*	NM_020702.3:c.[1318G>A;1189T>A] p.[(Ala440Thr);(Tyr397Asn)]	–	Hom	No	P	Novel
15	*SLC20A2*	NM_006749.4:c.380del p.(Leu127Argfs*44)	–	Het	Yes	P	Novel
16	Unknown	–	–	–	–	P	–
17	*MYORG*	NM_020702.3:c.674T>C p.(Leu225Pro)	NM_020702.3:c.862G>T p.(Asp288Tyr)	Comp‐Het	Yes	P	Novel
18	*MYORG*	NM_020702.3:c.1903C>A p.(Arg635Ser)	NM_020702.3:c.727G>T p.(Val243Leu)	Comp‐Het (?)	Yes (brother)	P	Novel
19	*MYORG*	NM_020702.3:c.674T>C p.(Leu225Pro)	NM_020702.3:c.862G>T p.(Asp288Tyr)	Comp‐Het (?)	No	P	Novel
20	*SLC20A2*	NM_001257180.2:c.1196A>C p.(His399Pro)	–	Het		P	Rubino *et al*. 2017 [30]

Patients 2 and 12 belong to the same family, as well as 3 and 4; all other patients are unrelated.

Comp‐Het, compound heterozygous variants; F, family member; Het, heterozygous variant; Hom, homozygous variant; P, proband.

**Table 2:** PFBC patients' clinical and radiological data. Age at biopsy and disease duration are reported in years.

Patient No.	Gene	Gender	Age at biopsy (y)	Family history	Motor phenotype	Non‐motor phenotype	Disease duration (y)	Calcification site	TCS
1	*PDGFB*	F	70	P	P, D	H, N (anxiety, depression)	16	BG, D, T	20
2	*MYORG*	F	69	P	P, C	MCI	6	BG, D, WM, T	42
3	*SLC20A2*	M	60	P	A	–	0	BG, D, WM, T	36
4	*SLC20A2*	M	54	P	A	–	0	BG, D, T	20
5	*MYORG*	M	44	N	D	N (anxiety, OCD), MCI	6	BG, D, WM, T	20
6	*SLC20A2*	F	49	N	A	–	0	BG, T	16
7	*MYORG*	F	55	N	P, C	MCI, N (depression)	5	BG, D, WM, T, C, B	61
8	Unknown	M	69	P (dem)	P	–	16	BG, D	14
9	*XPR1*	F	66	N	A	–	0	BG, D	14
10	*PDGFRB*	M	56	N	A	–	0	BG, D, WM, T	31
11	*SLC20A2*	F	53	P	A	–	0	BG	6
12	*MYORG*	M	68	P	P, C	N (depression), MCI	15	BG, D, WM, T, C	48
13	*SLC20A2*	F	39	P	D (tremor)	N (anxiety)	5	BG	6
14	*MYORG*	F	57	N	P, C	N, MCI, S	3	BG, D, WM, T, C	50
15	*SLC20A2*	F	49	P	D	N (anxiety), H	7	BG, D, T	15
16	Unknown	F	41	N	D	N (anxiety, depression), H, M	3	BG, T	12
17	*MYORG*	M	66	N	C	N (anxiety)	4	BG, D, WM	22
18	*MYORG*	M	56	P	A	N (depression), H	1	BG, D, T, WM	22
19	*MYORG*	M	56	N	C	N (anxiety), M	4	BG, D, T, WM, C, B	58
20	*SLC20A2*	F	49	N	P, C	N (depression), M, H	3	BG, D, T	16

Family history: N, negative; P, positive for PFBC; P (dem), positive family history for dementia with unknown CT scan. Motor phenotype: A, asymptomatic; C, cerebellar signs/ataxia; D, dystonia; P, parkinsonism. Non‐motor phenotype: H, headache; MCI, mild cognitive impairment; N, neuropsychiatric disturbances; OCD, obsessive‐compulsive disorder; S, seizures. Calcification site: B, brainstem; BG, basal ganglia; C, cerebral cortex; D, cerebellar dentate nuclei; T, thalami; TCS, total calcification score; WM, white matter.

**Figure 1:**
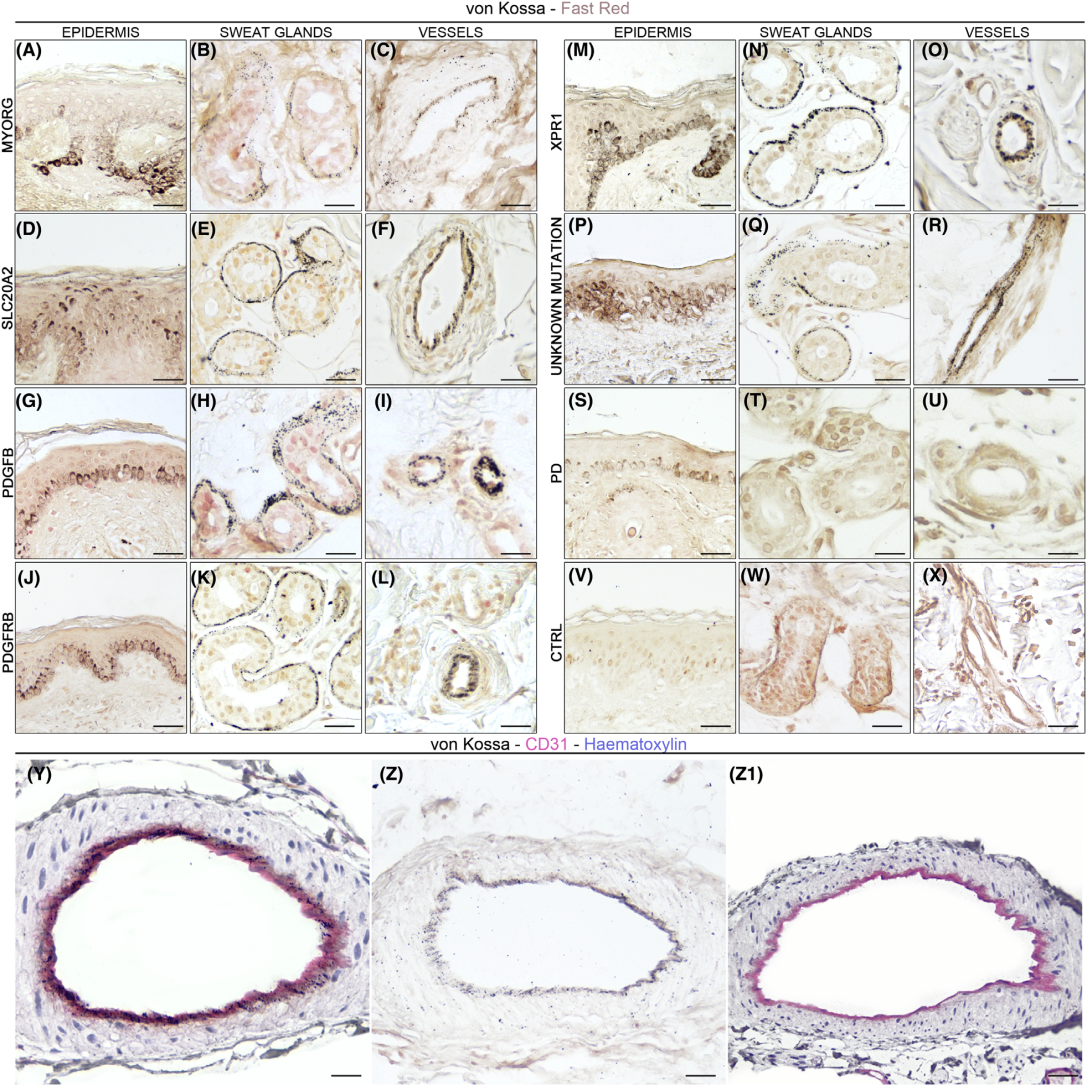
Calcium‐phosphate deposits in skin biopsies differentiate PFBC patients from Parkinson's disease (PD) patients and healthy controls (HC). (A–X) columns indicate the anatomical structures of interest (epidermis, sweat glands, blood vessels); rows identify single patients carrying different mutated genes, and one representative PD and HC case. (Y–Z1) colocalization between CD31 (magenta) and argyrophilic grains (black) in the blood vessels of PFBC patients.

In skin biopsies of 17/20 PFBC patients, small (0.2–0.5 μm) granular argyrophilic calcium‐phosphate deposits were identified in the intimal layer, and particularly in proximity to the basal lamina and within the cytoplasm of endothelial cells, in dermal capillaries and arterioles (Fig. 1). This pattern of basal lamina argyrophilic calcium‐phosphate grains was detected only in a fraction of total skin vessels (mean = 0.34 ± 0.1; average vessel profile count = 26 ± 4), and was consistently more frequent in deeper dermal arterioles (deep derma, reticular derma and dermo‐hypodermic junction) compared to superficial subepidermal capillaries (subepidermal and papillary derma).

A similar pattern was detected in the basement membrane of dermal sweat glands in 19/20 PFBC subjects: a fraction of total sweat gland adenomeres presented argyrophilic grains, but no distinct anatomical distribution of von Kossa+ secretory coils was detected (Fig. 1B–W). Sebaceous glands annexed to hair follicles did not present argyrophilic granular deposits.

Furthermore, in all PFBC patients, argyrophilic reaction of the basal layer of the epidermis was detected, varying from fine granular deposits to compact argyrophilic staining (Fig. 1A–V).

In neither PD (0/10) nor control subjects (0/7) (Fig. 1W,X), argyrophilic granular deposits were detected in sweat gland coils or the vascular wall. Similarly to PFBC cases, sebaceous glands annexed to hair follicles did not present argyrophilic granular deposits. In the basal layer of the epidermis, mild argyrophilic granular deposits were detected in a portion of subjects in both PD (6/10) and control group (4/7), likely representing cross‐reactivity with melanin pigmentation.

CD31 immunoperoxidase staining was detected for endothelial cells and pericytes in the intima of blood vessels, as well as in the basement membrane of sweat glands. In PFBC skin specimens, argyrophilic granular deposits partially colocalized with CD31+ structures (Fig. 1Y,Z); in particular, argyrophilic grains colocalized with CD31+ endothelial cell cytoplasm, but they were also found below and around endothelial cells, within the basal lamina. Granular deposits in the basal layer of the epidermis were also CD31‐.

## Discussion

In this study, we detected distinct patterns of calcium‐phosphate deposits in skin biopsies of PFBC patients, with variants in different known genes, via histopathology and light microscopy. Our findings indicate that, unlike in controls and PD, PFBC patients present granular argyrophilic calcium‐phosphate deposits in the basal lamina and the cytoplasm of CD31+ endothelial cells and pericytes, as well as in the basement membrane of sweat glands. Similarly to our findings, three PFBC patients with variants in *PDGFB*, *PDGFRB* and *XPR1* genes were previously reported to display microangiopathy and microcalcifications in the basal lamina in skin biopsies evaluated via electron microscopy.^18^, ^19^ In particular, *PDGFRB* variant carriers presented basal lamina thickening and fragmentation with microcalcifications within and around the pericytes, whereas the *XPR1* variant carrier presented microcalcifications within the cytoplasm of pericytes of the capillaries underneath the basal lamina.

In our study, light microscopy revealed the same pattern of calcium‐phosphate deposits in 17/20 PFBC patients, regardless of the underlying mutated gene, gender, clinical status (symptomatic vs asymptomatic) and disease duration and severity in manifesting subjects. In 3/20 patients, calcification patterns differed, being evident only in the basal layer of sweat gland coils, but not in the vascular wall. In these patients, skin biopsies suffered from lower sampling depth, with mostly reticular derma, and not deep derma, available for inspection. Considering the richness of vasculature in the deeper parts of the derma, this could have prevented the identification of calcium deposition at this level and suggests that multiple skin biopsies from the same patient, with adequate sampling depth, should be analyzed to increase the sensitivity of this staining/methods, as suggested in other degenerative diseases such as PD.^31^ Whether these findings reflect a common pattern of calcium‐phosphate deposition, regardless of genetics and affected molecular pathway, or could be attributed to the lower subcellular resolution of the technique used compared to electron microscopy, remains to be determined. Notably, while calcium‐phosphate deposits do not appear to significantly differ within the PFBC group (possibly due to the limited sample size of each genetic subgroup), shared patterns of grain deposition may suggest a common underlying pathophysiology. Animal and cellular models of the disease suggest that, through different pathways, functional disruption of the neurovascular unit, pericyte deficiency and endothelial alterations occur, subsequently leading to an osteogenic environment and neurodegeneration in specific brain areas.^16^, ^17^ In the skin, the basement membrane and the basal lamina appear to represent the preferential site of calcium‐phosphate deposition, without detectable signs of microangiopathy or vascular pathology. The vascular basal lamina is a specialized form of ExtraCellular Matrix (ECM) assembled by pericytes and endothelial cells and found between these cells. The basement membrane of sweat glands represents the main site anchoring epithelial cells to the surrounding connective tissue. Both structures are involved in angiogenesis and in regulating endothelial cell differentiation, while also acting as a mechanical barrier. Whether the cause or consequence of the disease, the detection of calcium‐phosphate deposits in basal laminae and endothelial structures in PFBC patients, but not controls, supports the notion of (neuro)vascular and barrier dysfunction. Lastly, confirmation of calcium‐phosphate deposits in structures outside of the central nervous system supports the notion of PFBC as a systemic disease, rather than a brain‐only entity. Given the shared ectodermal origin of the skin and the nervous system, which may represent an embryologically determined vulnerability factor leading to similar manifestations in terms of (neuro)vascular dysfunction in PFBC, it is currently unknown whether other organs may be affected by calcium‐phosphate deposits, and if this may turn into any clinical, diagnostic or prognostic significance. If the skin and the brain of PFBC patients share similar pathophysiological mechanisms, as suggested by our findings, skin biopsy may represent an intriguing model for the minimally invasive study of PFBC, an additional diagnostic tool or a potential biomarker to assess the efficacy of future therapies to reduce calcium deposition and the neurodegenerative processes leading to clinical manifestations.

We acknowledge that not all PFBC subjects showed the same results; this could be due to the sampling method, with scarce representation of deep dermal layers containing blood vessels in the 3/20 negative samples, or to different biological consequences of specific variants. For example, patient 13 carried an *SLC20A2* variant that was previously reported as a “mild” variant leading to less extensive brain calcifications affecting only the striatum,^29^ as in our case, hence this could explain the absence of extensive calcifications in the skin of this subject.

Future studies should be aimed at assessing if calcium‐phosphate deposition in the skin vasculature and sweat glands is a specific PFBC peripheral signature or may be present in other human diseases featuring primary or secondary basal ganglia calcification. This would clarify if skin biopsies may also have a role in the differential diagnosis of these diseases in a clinical setting since CT scan features alone can often be insufficient to distinguish among them.

Moreover, the potential relationship between peripheral calcification and disease duration, genetic status and clinical‐radiological features remains to be explored in larger cohorts.

## Funding Information

No funding was received towards this work.

## Conflict of Interest

The authors report no competing interests.

## Author Contributions

AE, GB, and MC contributed to the conception and design of the study. AE, GB, MC, AT, FC, AP, CB, and LS contributed to the acquisition and analysis of data. AE, GB, and AT contributed to preparing the figures; AE, GB, AA, and MC contributed to the draft and critical revision of the text.

## Supporting information


Appendix S1.

## Data Availability

Data (including images) supporting the findings of this study are available from the corresponding author, upon reasonable request.
