# Supplementary material for: Skin calcium deposits in primary familial brain calcification: A novel potential biomarker
Source: Ann Clin Transl Neurol. 2025 Feb 11;12(4):737–45. doi: 10.1002/acn3.52304 (PMC12040503; doi:10.1002/acn3.52304)
Supplement: Supplementary file 1 — Appendix S1. [file ACN3-12-737-s001.docx]

**Skin calcium deposits in Primary Familial Brain Calcification: a novel potential biomarker**

**Annals of Clinical and Translational Neurology**

Aron Emmi, PhD^1,2,3,*^; Giulia Bonato, MD^2,3,*^; Aleksandar Tushevski, MSc^1^; Cinzia Bertolin, PhD^4^; Francesco Cavallieri, MD PhD^5^; Andrea Porzionato, MD PhD^1^; Angelo Antonini, MD PhD ^2,3^; Leonardo Salviati, MD PhD^4,6^; Miryam Carecchio, MD PhD^2,3^

** These authors contributed equally to this manuscript as first authors*

*^1^ Institute of Human Anatomy, Department of Neurosciences, University of Padova, 35129 Padova, Italy*

*^2^ Parkinson and Movement Disorders Unit, Centre for Rare Neurological Diseases (ERN-RND), Department of Neuroscience, University of Padova, 35128 Padova, Italy*

*^3^ Center for Neurodegenerative Disease Research (CESNE), University of Padova, 35128 Padova, Italy*

*^4^ Clinical Genetics Unit, University Hospital of Padova, 35128 Padova, Italy.*

*^5^ Neurology Unit, Neuromotor and Rehabilitation Department, Azienda USL-IRCCS di Reggio Emilia, 42123 Reggio Emilia, Italy*

*^6^ Clinical Genetics Unit, Department of Women's and Children's Health, University of Padova, 35128 Padova, Italy.*

**Corresponding author:**

Miryam Carecchio, MD, PhD

Department of Neuroscience, Via N. Giustiniani 5, 35128 Padova, Italy

Email: [miryam.carecchio@unipd.it](mailto:miryam.carecchio@unipd.it)

**Supplementary Material**

**Supplementary List**. List of genes analyzed in this work.

*AARS1, ABCA1, ABCC6, ACTA2, ACTB, ADA2, ADAR, ADCY5, AFG3L2, ALAD, ALAS2, ALS2, AMT, ANG, ANO10, ANO3, ANXA11, APOA1, APOB, APOE, APP, APTX, ARFGEF3, ARX, ATAD1, ATM, ATP13A2, ATP1A2, ATP1A3, ATP5B, ATP6AP2, ATP7A, ATP7B, AUTS2, BCAP31, C9ORF3, C10ORF2, C19ORF12, CACNA1A, CACNA1B, CACNB4, CAMK2B, CCM2, CFAP410, CHCHD10, CHCHD2, CHD6, CHD8, CHMP2B, CLDN5, CLPX, CMPK2, COASY, COL4A1, COL4A2, COQ4, COQ8A, CP, CPOX, CRAT, CSF1R, CSMD1, CST3, CTC1, CTSA, CYP27A1, DCAF17, DCTN1, DDC, DHCR24, DHDDS, DHX30, DNAJC12, DNAJC13, DNAJC5, DNAJC6, DRD2, ECHS1, EIF2AK2, EIF4G1, ENPP1, ERBB4, FA2H, FBXO7, FECH, FIG4, FLVCR1, FOXC1, FOXF2, FOXG1, FRRS1L, FTL, FUS, FXN, GBA, GCH1, GCSH, GLA, GLB1, GLDC, GLE1, GLRA1, GLRB, GM2A, GNAL, GNAO1, GNE, GPR88, GRID2, GRIN1, GRM1, GRN, GUCY1A1, HIBCH, HMBS, HNRNPA1, HPCA, HTRA1, IFIH1, IMPDH2, ITM2B, JAM2, JAM3, KCNA1, KCNK18, KCNMA1, KCNN1, KCTD17, KIAA1244, KMT2B, KRIT1, LDLR, LDLRAP1, LINGO4, LPL, LRP10, LRRK2, MAPT, MATR3, MECR, MED27, MICU1, MORC2, MRE11A, MSL3, MTPAP, MTTP, MYORG, NAA60, NEK1, NKX2-1, NOTCH3, NPC1, NPC2, NT5E, NUP62, OCLN, OPTN, PAK1, PANK2, PARK7, PCDH12, PCSK9, PDCD10, PDE10A, PDE2A, PDE8B, PDGFB, PDGFRB, PFN1, PGK1, PINK1, PITX2, PLA2G6, PNKD, PNKP, PNPT1, POLG, PPOX, PPP2R5D, PRKN, PRKRA, PRNP, PRRT2, PSEN1, PSEN2, RAB39B, REPS1, RNASEH2A, RNASEH2B, RNASEH2C, RNASET2, SACS, SAMHD1, SCN1A, SCN2A, SCN8A, SCP2, SERPINI1, SETX, SGCE, SIGMAR1, SIL1, SLC16A2, SLC18A2, SLC19A3, SLC1A3, SLC20A2, SLC25A19, SLC2A1, SLC2A10, SLC30A10, SLC39A14, SLC6A3, SLC6A5, SMPD1, SNCA, SNCB, SOD1, SPG11, SPR, SQSTM1, SRY, STN1, STXBP1, SUCLA2, SYNE1, SYNJ1, SYT1, SYT14, TAF1, TARDBP, TBC1D24, TBK1, TDP1, TECPR2, TH, THAP1, TIMM8A, TMEM240, TOR1A, TPK1, TREM2, TREX1, TSPOAP1, TTPA, TTR, TUBA4A, TUBB4A, TWNK, TYROBP, UBQLN2, UBTF, UCHL1, UNC13A, UQCRC1, UROD, UROS, USP18, VAC14, VAPB, VCP, VPS11, VPS13A, VPS13C, VPS13D, VPS16, VPS35, VPS41, WDR45, WDR73, XK, XPR1, YY1, ZEB2, ZMYND11, ZNF532.*

Additional genes (if clinical suspicion): *DKC1, TINF2, TERC, TERT, NHP2, NOP10, WRAP53, RTEL1, CA2, FOLR1, SLC46A1, MTHFR, DHFR, MTFD1, DDB2, ERCC1, ERCC2, ERCC3, ERCC4, ERCC5, ERCC6, ERCC8, GTF2H5, MPLKIP, POLH, XPA, XPC, PSMB8, DOCK6, FAM20C, MGP, AP1S2, ISG15, ACP5, FARSA, FARSB, NRROS, CYP2U1, GNAS, PRKAR1A, PDE4D, PDE3A, mtDNA mutations.*

**Supplementary Table S1.** Variants in PFBC related genes found in this study, with ACMG-AMP classification, items applied for classification and MAF (maximum allele frequency).

| **Patient No.** | **Gene** | **Mutation** | **ACMG -AMP** | **ACMG Items Applied** | **MAF GnomAD 4.1** |
| --- | --- | --- | --- | --- | --- |
| **1** | *PDGFB* | NM_002608.2:c.298C>T p.(Arg100Cys) | 4 | PM2, PP3, PP1, PP4, PP5^[25]^ | 6.20e-7 |
| **2, 12** | *MYORG* | NM_020702.3:c.686G>A p.(Trp229*) | 4 | PVS1, PM2, PM3, PP1, PP4 | 6.21e-7 |
| **2, 12, 14** | *MYORG* | NM_020702.3:c.1189T>A p.(Tyr397Asn) | 4 | PM2, PM3, PP1 (patient 2 and 12), PP4 | 6.20e-7 |
| **2, 12, 14** | *MYORG* | NM_020702.3:c.1318G>A p.(Ala440Thr) | 4 | PM2, PM3, PP1 (patient 2 and 12), PP4 | 6.23e-7 |
| **3, 4** | *SLC20A2* | NM_006749.4:c.84T>A p.(Asp28Glu) | 4 | PM2, PM5, PP3, PP4, PP1 | 0 |
| **5** | *MYORG* | NM_020702.3:c.1270_1277dup p.(Trp426Cysfs*11) | 5 | PVS1, PM2, PM3, PP4, PP5^[26]^ | 6.84e-6 |
| **6** | *SLC20A2* | NM_001257180.2:c.212G>A p.(Arg71His) | 4 | PP3, PM2, PP4, PP5^[27,28]^ | 1.86e-6 |
| **7** | *MYORG* | NM_020702.3:c.1321del p.(Arg441Alafs*65) | 4 | PVS1, PM2, PM3, PP4 | 0 |
| **7** | *MYORG* | NM_020702.3:c.1841T>C p.(Leu614Pro) | 4 | PM2, PP3, PM3, PP4 | 6.22e-7 |
| **9** | *XPR1* | NM_004736.4:c.194A>G p.(Glu65Gly) | 4 | PM2, PP3, PP2, PP4 | 0 |
| **10** | *PDGFRB* | NM_002609.3:c.2051G>T, p.(Cys684Phe) | 4 | PM2, PP3, PP4 | 0 |
| **11** | *SLC20A2* | NM_001257180.2:c.1472A>G p.(Gln491Arg) | 4 | PM2, PP3, PP1, PP4 | 0 |
| **13** | *SLC20A2* | NM_006749.4:c.541C>T p.(Arg181Trp) | 4 | PM2, PP3, PP1, PP4, PP5^[29]^ | 9.31e-6 |
| **15** | *SLC20A2* | NM_006749.4:c.380del p.(Leu127Argfs*44) | 4 | PVS1, PM2, PP1, PP4 | 0 |
| **17, 19** | *MYORG* | NM_020702.3:c.674T>C p.(Leu225Pro) | 4 | PM2, PP3, PM3, PP4 | 4.35e-6 |
| **17, 19** | *MYORG* | NM_020702.3:c.862G>T p.(Asp288Tyr) | 4 | PM2, PP3, PM3, PP4 | 1.24e-6 |
| **18** | *MYORG* | NM_020702.3:c.1903C>A p.(Arg635Ser) | 4 | PM2, PP3, PP4, PM3, PP1 | 1.24e-5 |
| **18** | *MYORG* | NM_020702.3:c.727G>T p.(Val243Leu) | 4 | PM2, PP4, PM3, PP1 | 6.20e-7 |
| **20** | *SLC20A2* | NM_001257180.2:c.1196A>C p.(His399Pro) | 4 | PS4, PM2, PP4, PP5^[30]^ | 1.24e-6 |
